# Integrated transcriptomic and metabolomic analyses reveal the molecular mechanism of flower color differentiation in *Orychophragmus violaceus*

**DOI:** 10.3389/fpls.2025.1509120

**Published:** 2025-02-14

**Authors:** Yubin Shi, Zixuan Wang, Zhuangzhuang Yan, Jianfeng Liu, Jun Zhang, Guixia Liu

**Affiliations:** ^1^ School of Life Sciences, Hebei University, Baoding, China; ^2^ School of Life Sciences, Institute of Life Science and Green Development, Hebei University, Baoding, China; ^3^ College of Life Science, Engineering Research Center of Ecological Safety and Conservation in Beijing–Tianjin–Hebei (Xiong’an New Area) of Ministry of Education (MOE), Hebei University, Baoding, China

**Keywords:** *Orychophragmus violaceus*, flower color, molecular mechanism, anthocyanin, transcriptomic and metabolomic

## Abstract

**Introduction:**

*Orychophragmus violaceus* is a popular horticultural plant because of its bright purple flowers that are commonly found in parks and green belts. However, three flower colors (purple, light purple, and white) were observed in the wild-type *O. violaceus*. The molecular mechanism underlying the formation of these intriguing flower colors remains unknown.

**Methods:**

Here, we combined metabolomics and transcriptomics to identify a pathway cascade leading to anthocyanin biosynthesis associated with flower color formation in *O. violaceus*.

**Results and discussion:**

A total of 152 flavonoid metabolites were identified based on metabolomic data, most of which were quercetin and kaempferol. Comparative analysis of the metabolites among the three flower samples revealed that two anthocyanins, peonidin-3-glucoside and delphinidin 3-(6’’-malonyl-glucoside), are the pigments most likely responsible for the coloration of the petals of *O. violaceus*. Subsequent transcriptomic analysis revealed 5,918 differentially expressed genes among the three groups of flowers, 87 of which encoded 13 key enzymes in the anthocyanin biosynthetic pathway. Moreover, the high expression of two transcription factors, *OvMYB* and *OvbHLH*, in purple flowers suggests their role in the regulation of anthocyanin biosynthesis. By integrating metabolomic and transcriptomic data, *OvANS*, which encodes anthocyanidin synthase, was significantly upregulated in purple flowers. *OvANS* is the enzyme responsible for the transformation of colorless leucoanthocyanidins to colored anthocyanidins. This study provides novel insights into the molecular mechanism of flower color development in *O. violaceus*, laying the foundation for flower color breeding.

## 1 Introduction

The flower colors of various plants in nature span the entire color spectrum of both humans and pollinators (Rezende et al., 2020). Plants of different species often differ in flower color, and even the same species differs in geographic and temporal scales (Trunschke et al., 2021). Flower color not only affects the interaction between plants and pollinators but also serves as an important quality determinant for ornamental plants (Zhou et al., 2023; Sun et al., 2021; Wang et al., 2024a). Many colorful species, such as *Chrysanthemum*, *Cymbidium*, and *Malus halliana*, have been selected as ornamental plants (Tang et al., 2022; Li et al., 2021a; Han et al., 2020).

The formation of flower color is determined mainly by the type and content of colored secondary metabolites such as anthocyanins, carotenoids, and betaine in flower tissue (Jiang et al., 2020; Guo and Qiu, 2013; Tanaka et al., 2008). Among these three pigments, carotenoids are fat-soluble chemicals that can appear yellow to red and are widely distributed in seed plants and are involved in photosynthesis (Guan et al., 2023; Sun et al., 2024; Wang et al., 2018). Betaines are water-soluble chemicals that appear yellow to red and occur only in the order Caryophyllales (Tomizawa et al., 2021). Anthocyanins, which are composed of flavones and aglycones, are soluble in water and are reported to be the dominant metabolites that determine flower color (Wang et al., 2024b; Li et al., 2022). Anthocyanins enable flowers to exhibit a broad spectrum of colors, from orange and red to purple and blue (Zhang et al., 2022; Guo et al., 2019; Xiao et al., 2023). There are six types of anthocyanins, including pelargonidin (Pg), cyanidin (Cy), delphinidin (Dp), peonidin (Pn), petunidin (Pt), and malvidin (Mv) (Tanaka and Brugliera, 2013; Zhang et al., 2014). The types of anthocyanins vary among different species of plants and are the main factors affecting different flower colors. In addition, flavone and flavonol derivatives are responsible for copigmentation, endowing plants with unlimited color variation (Liu et al., 2020).

*Orychophragmus violaceus* (family Brassicaceae) is an annual or biennial herb, also known as the ‘February orchid’ for its flowering phase in February and is widely distributed in Northeast China and North China. *O. violaceus* is mainly used as an oil-producing crop and ornamental plant in China (Wang et al., 2014; Guo et al., 2022). Purple flowers are the main reason for its popularity as a horticultural plant. However, three flower colors (purple, light purple, and white) were observed in the wild-type *O. violaceus*. To date, the molecular mechanism underlying purple flower formation in *O. violaceus* remains unknown. Anthocyanins have been proven to be the dominant constituents of the purple pigments of flowers in several plants, such as *Plumbago auriculata*, *Scutellaria baicalensis*, and Monkeyflowers (*Phrymaceae*) (Li et al., 2021b; Hu et al., 2023; Grossenbacher et al., 2021). Additionally, Honda et al. (2005) reported that the purple petals of *O. violaceus* are rich in anthocyanins. Therefore, we speculated that anthocyanins might contribute to the development of purple flowers in *O. violaceu*s. In this study, three groups of flowers (purple, light purple, and white) were investigated to explore the differences in anthocyanin metabolism and transcription of related biosynthetic genes. Here, we combined transcriptomic and metabolomic data of *O. violaceus* to determine whether anthocyanins are the dominant pigments involved in purple petal formation.

## 2 Materials and methods

### 2.1 Plant materials

*O. violaceus* samples were taken from the test site on Hehua Road in Baoding City, Hebei Province (115.57°E, 38.85°N). Three distinct flower colors (purple, light purple, and white) were collected at the full bloom stage on 8 April 2023, and named OvP, OvLP, and OvW, respectively. The colors of the flowers were compared via CIELAB analysis using a spectrophotometer, and each flower was tested three times. All samples were frozen in liquid nitrogen and stored at −80°C for metabolomic and transcriptomic sequencing studies.

### 2.2 Determination of relative anthocyanin content

The total anthocyanin content was measured using the method reported by Jiang et al. (2020). A 0.100 g fresh flower sample was mixed with 1.0 ml of the extract (methanol:hydrochloric acid = 99.9:0.1) and ground. The mixture was ultrasonically shaken for 30 s and centrifuged at 12,000×*g* for 2 min at 4°C. The absorbance was measured at 530 nm and 600 nm. The results are expressed as units g{sp}−1{/sp} FW.

### 2.3 Metabolite extraction

A solid sample of 100 mg and 400 µL of extract solution (methanol:water = 4:1(v:v)) containing four internal standards (L-2-chlorophenylalanine (0.02 mg/mL), etc.) were added in a 2 mL centrifuge tube with a diameter of 6 mm abrasive bead. The sample solution was ground in a frozen tissue grinder for 6 min (−10 °C, 50 Hz) and then extracted by ultrasound at a low temperature for 30 min (5 °C, 40 kHz). The sample was placed at −20°C for 30 min, centrifuged for 15 min (4 °C, 13,000*g*), and the supernatant was transferred to an injection vial with internal intubation for machine analysis. The flowers of the three colors were repeated three times, and a total of nine samples were collected. The supernatant of each sample (20 µL) was mixed as a quality control sample. The instrument platform for this LC–MS analysis was the UHPLC-Q Exactive system of Thermo Field’s ultra-high-performance liquid chromatography tandem Fourier Transform mass spectrometry (Majorbio, Shanghai).

#### 2.3.1 Chromatographic conditions

The chromatographic column was an ACQUITY UPLC BEH C18 column (100 mm × 2.1 mm i.d., 1.7 µm; Waters, Milford, USA), mobile phase A consisted of 2% acetonitrile water (containing 0.1% formic acid), and mobile phase B consisted of acetonitrile (containing 0.1% formic acid). The sample size was 3 μL, and the column temperature was 40 °C.

#### 2.3.2 Mass spectrum conditions

The sample quality spectrum signal was collected in positive and negative ion scanning modes, and the quality scanning range was 70 m/z–1,050 m/z. Ion spray voltage: positive ion voltage 3,500 V, negative ion voltage −3,000 V, sheath gas flow rate 50 arb, auxiliary gas flow rate 13 arb, ion source heating temperature 450 °C, 20–40–60 V cyclic collision energy, Full MS resolution 70,000, and MS^2^ resolution 17,500 (Yang et al., 2024).

### 2.4 Metabolite identification and analysis

After completion of the computer, the LC–MS raw data were imported into the metabolomics processing software Progenesis QI (Waters Corporation, Milford, USA) for processing, and a data matrix of retention time, mass-charge ratio, and peak intensity was obtained. Then, the data matrix was filtered for low-quality peaks, missing values were removed, vacant values were filled, normalization, QC sample relative standard deviation (RSD) assessment, data transformation, and other preprocessing was performed to standardize the data structure. Second, the ropls package (Version1.6.2) in R language was used to conduct principal component analysis (PCA) and orthogonal least partial square discriminant analysis (OPLS-DA) on the pre-processed data matrix, and the stability of the model was evaluated using seven cycles of interactive verification. Use http://www.hmdb.ca/, https://metlin.scripps.edu/ and others public database and self-built database to identify the metabolite search library. The selection of metabolites with significant differences was based on the variable weight value (VIP) obtained using the OPLS-DA model and the *P*-value of the Student’s t-test. Metabolites with VIP >1 and *P <*0.05 were identified as significantly different metabolites. Differences in metabolites by KEGG database (https://www.kegg.jp/kegg/pathway.html) for the metabolic pathways of annotation and differences in metabolites involved in pathways. Python software package scipy was used. Stats were used for pathway enrichment analysis, and the biological pathways most relevant to the experimental treatment were obtained using Fisher’s exact test.

### 2.5 RNA sequencing

The petals of nine freeze-dried samples were ground for RNA extraction using an MJZol Total RNA Extraction Kit (Majorbio, Shanghai, China). The concentration and purity of the extracted RNA were detected using Nanodrop2000. RNA integrity was measured using Biowest Agarose (Biowest, Spain), and RIN values were determined using Agilent2100. The mRNA was randomly fractured using a fragmentation buffer, and small fragments of approximately 300 bp were separated by magnetic bead screening. Six-base random hexamers were added to invert the mRNA template to synthesize the first cDNA, and the second strand was then synthesized to form a stable double-stranded structure. The End Repair Mix was added to the double strand to form a flat end, followed by the addition of nucleotide A to the 3’ end to join the Y-shaped joint. After adapter connection, the product was purified and the fragments were sorted. The sorted product was enriched by PCR (T100 Thermal Cycler, Bio-Rad, USA), and the final library was purified. Quantification was performed using Qubit 4.0, and bridge PCR amplification was performed using cBot to generate the clusters. Sequencing was performed using an Illumina NovaSeq 6000 instrument with a read length of 2 ∗ 150 bp. The data generated by sequencing were stored in Fastq format. Sequencing data quality control using software fastp (https://github.com/OpenGene/fastp), including the sequence of quality evaluation, deletion, low quality of the sequence (including joint series, low quality of reading section, N rate (N uncertain information base) high, and long for short sequences), and clean reads were retained for subsequent analysis.

### 2.6 Transcriptome data analysis

Sequencing data after quality control and filtering, using HiSat2 (http://ccb.jhu.edu/software/hisat2/index.shtml) and the reference genome alignment, for subsequent transcription of this assembly, expression quantity calculation and so on mapped data (reads), At the same time, the quality of the transcriptome sequencing results was evaluated. Using RSEM (http://deweylab.github.io/RSEM/), the expression levels of gene transcription and quantitative analysis, differentially expressed by DESeq2 (http://bioconductor.org/packages/stats/bioc/DESeq2) analysis, the threshold of differentially expressed genes was set to P-adjust values <0.05, fold change ≥2, and the BH multiple calibration method was used to correct. According to the Kyoto Encyclopedia of Gene and Genome (KEGG) pathway database, NCBI non-redundant (Nr) database, Swiss-Prot Protein Sequence Database (Swiss-Prot), Evolutionary Genealogy of Genes: Non-supervised Orthologous Groups (EggNOG), Gene Ontology (GO), and Protein Family Database (Pfam), annotated gene function. Differences in gene function of KEGG pathway enrichment were analyzed using the Python scipy software package (https://scipy.org/install/) (Young et al., 2010; Kanehisa et al., 2023).

### 2.7 qRT-PCR analysis

The extracted RNA was reverse-transcribed into cDNA using a Hiscript III RT SuperMix for qPCR (+gDNA wiper) reverse transcription kit (Vazyme, China), according to the manufacturer’s instructions. The Actin 7 gene (ID: OV05G038310) of *O. violaceus* was used as the internal reference gene. The ChamQ Universal SYBR qPCR Master Mix kit (Vazyme, China) was used to detect the relative expression levels of 13 genes related to the anthocyanin synthesis pathway. Gene-specific primers used are listed in **Supplementary Table S1**. Real-time fluorescence quantitative polymerase chain reaction (qRT-PCR) was performed using ABI Prism 7500 real-time fluorescence quantitative PCR system. Quantitative analyses were performed using the 2{sp}−ΔΔCT{/sp} method (Zhao et al., 2022).

### 2.8 Statistical analysis

Statistical analysis was performed using the SPSS Statistics 27 software (GraphPad Software, Inc.). Data are presented as the mean ± standard deviation (SD). The level of statistical significance was analyzed using the least significant difference test (p <0.05).

## 3 Results and discussion

### 3.1 Relative contents of total anthocyanins in the flowers of *O. violaceus*

**Figure 1:**
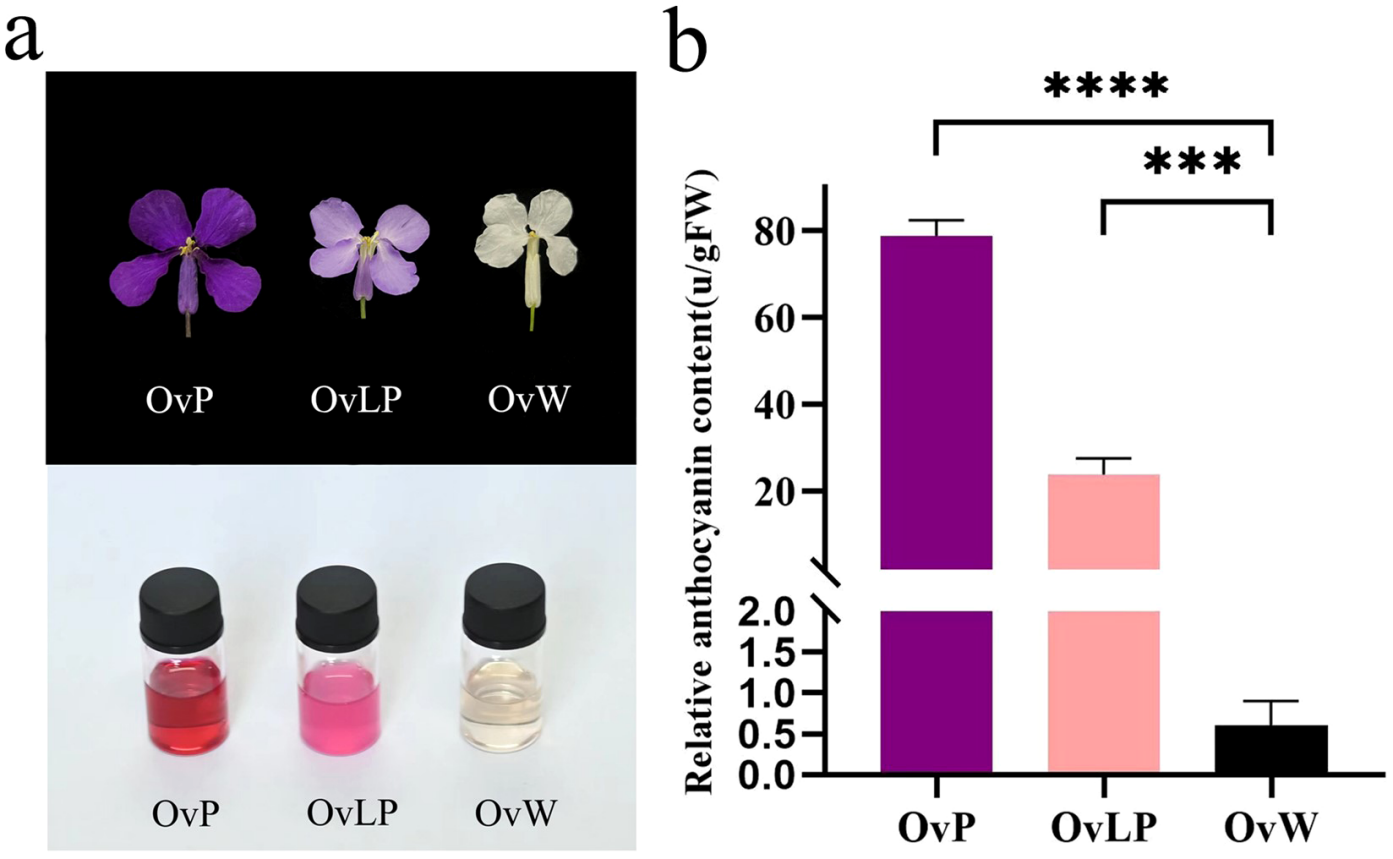
Comparison of flower phenotypes and relative anthocyanin contents in different flower colors of *O. violaceus*: **(A)** Comparison of mature petals and their anthocyanin extracts. **(B)** Relative content of total anthocyanins in white, light purple, and deep purple mature petals (asterisk (***) for *P <*0.001, (****) for *P <*0.0001).

Phenotypically, the flowers of the three colors displayed no observable differences in shape, but the OvW and OvLP flowers were smaller than the OvP flowers (**Figure 1A**). Intuitively, the pigment contents of OvP and OvLP were significantly greater than that of OvW. We selected three fully blooming flowers for anthocyanin measurement and reported that the anthocyanin content in OvP (~78.77 units/g FW) was significantly greater than that in OvLP (~23.83 units/g FW) and OvW (~0.60 units/g FW) (**Figure 1B**). These results suggest that total anthocyanin content might play an important role in the color of *O*. *violaceus*.

### 3.2 Metabolic differences in flavonoids

**Figure 2:**
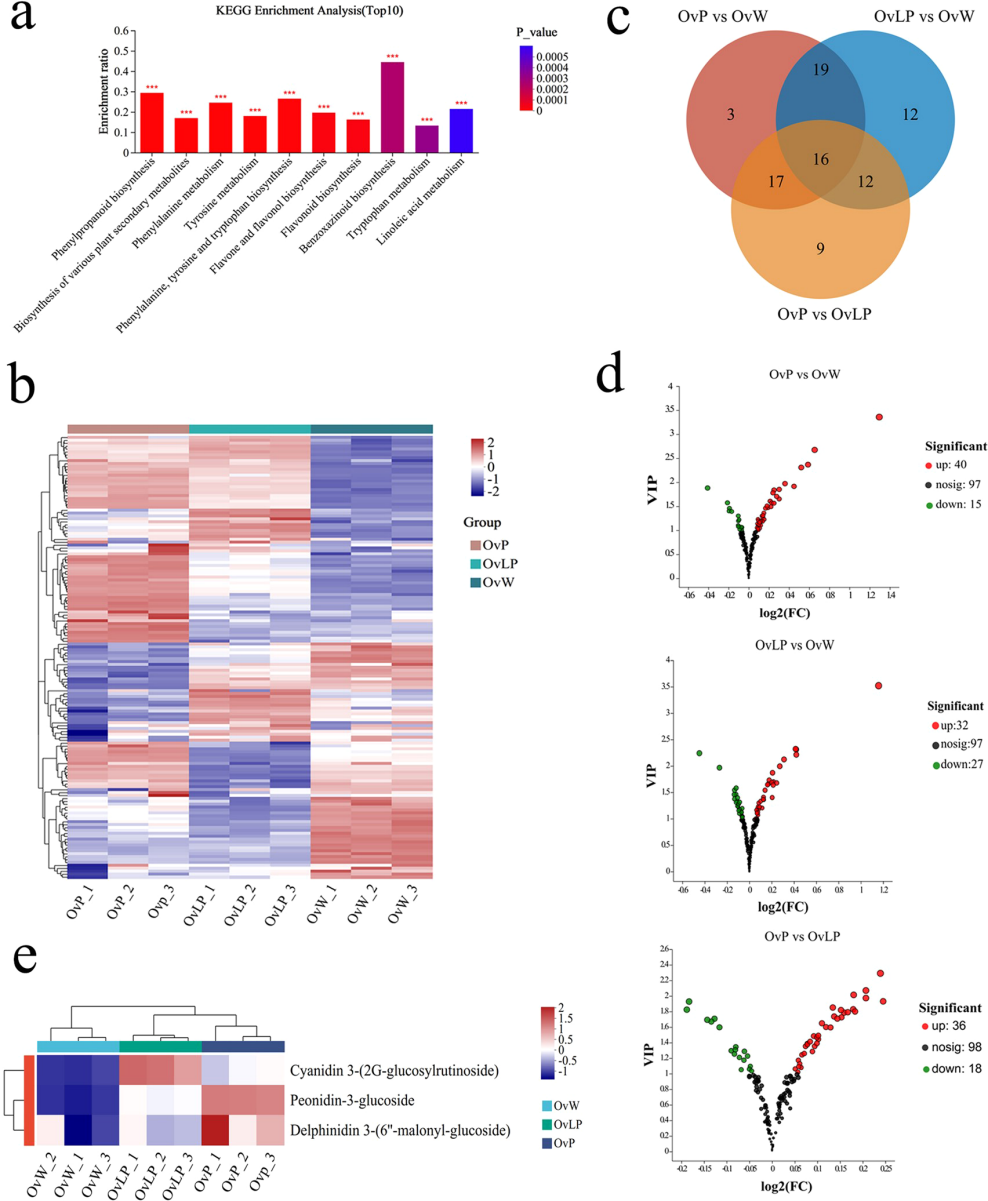
Metabolomic analysis of *O. violaceus*: **(A)** KEGG pathway enrichment histogram of the top 10 differentially accumulated metabolites. The asterisk (*) indicates a significant difference (***P <0.001, based on Duncan’s multivariate range test). **(B)** Stratified cluster heatmaps of the 152 flavonoid metabolites in each sample. **(C)** Venn diagrams and bar charts of the compositions of different groups of flavonoid metabolites. **(D)** Volcanic map of the differential isoflavone metabolites. **(E)** Clustering heat maps of three different accumulated anthocyanins.

To investigate the metabolic differences of flavonoids in flowers of the three colors, we performed untargeted metabolome analysis via LC−MS and established a high-capacity metabolic library. KEGG metabolic pathway enrichment analysis with a P value <0.05 indicated that metabolic processes associated with the biosynthesis of phenylalanine and flavonoids were enriched (**Figure 2A**). A total of 152 flavonoid metabolites were detected and classified into 11 types: anthocyanins (3), chalcones (3), flavanols (1), flavanones (2), flavones (9), flavonols (8), isoflavones (18), new flavonoids (1), flavone glycosides (94), other flavones (8), and flavanonols (5) (**Figure 2B**). Pairwise comparisons (OvP vs OvW, OvLP vs OvW, and OvP vs OvLP) revealed significant metabolic differences in 88 flavonoids, including 55 flavonoids in OvP vs OvW (up: 40, down: 15), 59 in OvLP vs OvW (up: 32, down: 27), and 54 in OvP vs OvLP (up: 36, down: 18) (VIP value >1) (**Figures 2C, D**). Additionally, three anthocyanins were identified in all the flower samples, including peonidin-3-glucoside, delphinidin 3-(6”-malonyl-glucoside), and cyanidin 3-(2G-glucosylrutinoside), which accumulated significantly in OvP and OvLP (**Figure 2E**). Notably, peonidin-3-glucoside and delphinidin 3-(6’’-malonyl-glucoside) were positively correlated with the degree of color of the three flower samples, suggesting that they might be the main components of the flower pigments of *O*. *violaceus*. The correlation between peonidin and plant petal color has also been characterized in *Rosa rugosa* (Zan et al., 2024).

### 3.3 Expression profile of structural genes involved in flavonoid biosynthetic pathways

Transcriptome sequencing was performed using the Illumina NovaSeq 6000 sequencing platform to analyze gene expression associated with anthocyanin metabolic pathways in the three groups of flower samples. A total of 71.09 Gb of clean data were obtained, with 94.44% of the bases having Q30 scores. For all the clean reads, the comparison rate with the reference genome (https://ngdc.cncb.ac.cn/search/?dbId=gwh&q=GWHBGBQ00000000&page=1) of *O. violaceus* ranged from 69.24% to 72.49%. BLASTX was used to retrieve all matched clean reads from the NR, SwissProt, Pfam, KEGG, GO, and eggNOG databases, along with functional annotations.

**Figure 3:**
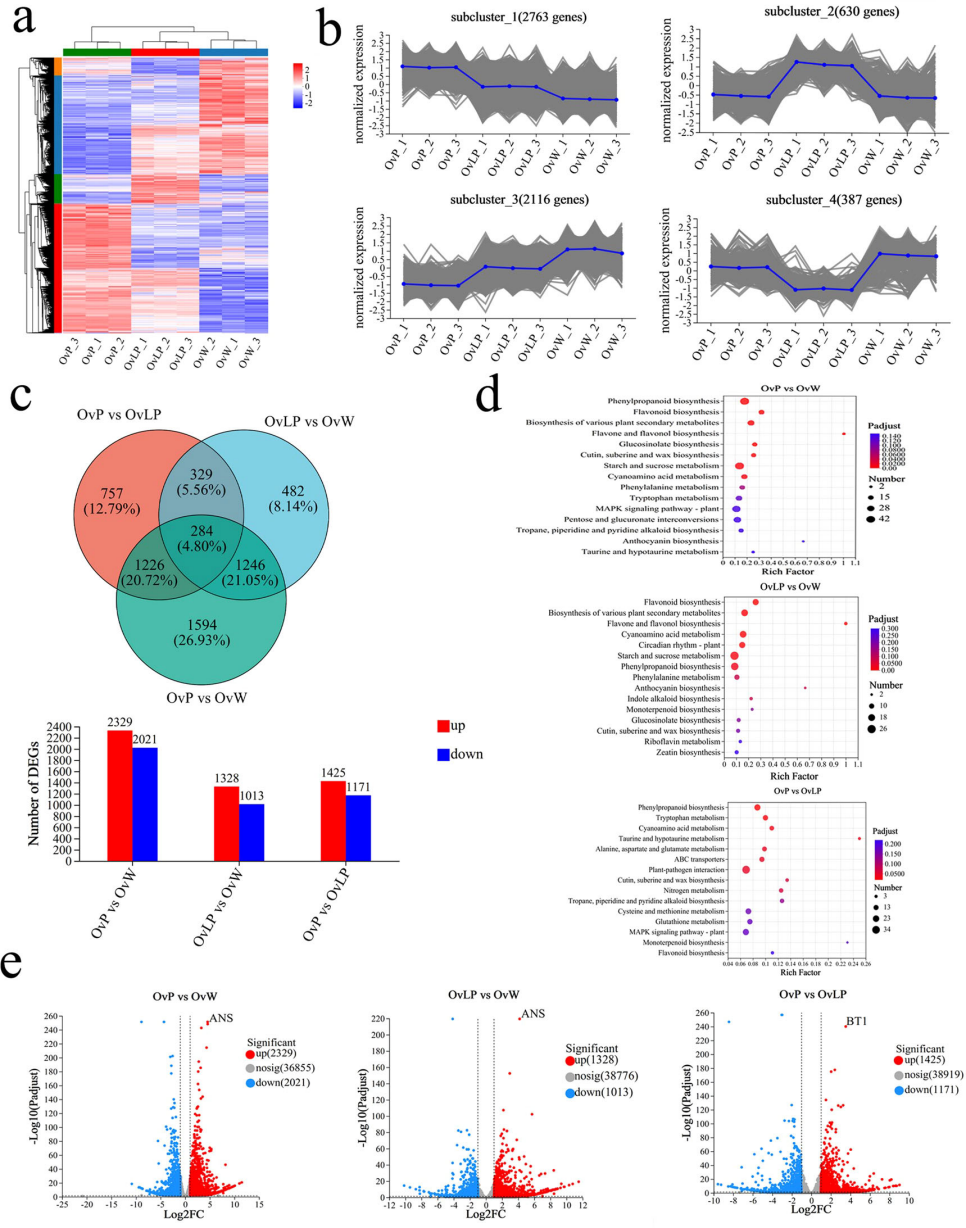
Transcriptomic analysis of *O. violaceus*: **(A)** Clustering heatmap of DEGs (5918). **(B)** DEG subcluster trend graph. **(C)** Venn diagram and histogram of DEGs. **(D)** Bubble diagram of the KEGG metabolic pathway enrichment in the three comparison groups. **(E)** Volcano map of differentially expressed genes.

According to the criteria of FDR <0.05, and |Log2FC| ≥1, 5,918 differentially expressed genes (DEGs) were identified in the three groups of flowers. Through cluster analysis of DEGs, tissue-specific transcriptomic maps of *O. violaceus* flowers were generated (**Figure 3A**). The expression index of cluster analysis was calculated using the fragments per kilobase and per million reads (FPKM) method. Hierarchical clustering analysis was performed on the DEGs, and four main subclustering trends, namely, purple upregulation, light purple upregulation, purple downregulation and light purple downregulation, were obtained (**Figure 3B**). The Venn diagram revealed 4,350, 2,341, and 2,596 DEGs in the flowers of the three groups: OvP vs OvW, OvLP vs OvW, and OvP vs OvLP, respectively. Among the DEGs, 2,329, 1,328, and 1,425 genes were upregulated, whereas 2,021, 1,013, and 1,171 genes were downregulated, respectively (**Figure 3C**). KEGG metabolic pathway enrichment analysis indicated that the DEGs were mainly enriched in flavone, flavonol, and anthocyanin biosynthetic pathways (**Figure 3D**). Analysis of transcriptome data revealed that 87 genes encoding 13 key enzymes, including *OvPAL* (6), *OvC4H* (3), *Ov4CL* (18), *OvCHS* (8), *OvCHI* (11), *OvF3H* (8), *OvF3’H* (5), *OvF3’5’H* (1), *OvDFR* (4), *OvANS* (1), *OvUFGT* (9), *Ov3MaT1* (2), and *OvMT* (11), were associated with the anthocyanin biosynthetic pathway. According to the expression results, *OvPAL*, *OvC4H*, *OvCHS*, *OvANS*, *OvUFGT* and most other structural genes in the anthocyanin biosynthetic pathway of *O*. *violaceus* were significantly upregulated in OvP and OvLP. Notably, *OvANS* (ID: Ov03G032130) was the most significantly upregulated DEG among the three flower types (**Figure 3E**). The gene transcription levels of *OvANS* in OvP and OvLP were 22 and 17 times greater than those in OvW, respectively (**Figure 3E**). The effect of *ANS* on flower color change has been verified in many species (Hu et al., 2023; Wu et al., 2023). Sequence alignment revealed that OvANS has presented 96.4%, 95%, and 94% amino acid identity with BjANS (*Brassica juncea*, ACH58397.1), RsANS (*Raphanus sativus*, ALH21136.1), and MiANS (*Matthiola incana*, AAB82287.1), respectively. Phylogenetic analysis revealed that OvANS is closely related to ANS proteins of *Thlaspi arvense* and *Brassica carinata* (**Supplementary Figure S2**).

Anthocyanin biosynthesis is positively regulated by members of the *MYB*, *bHLH*, and *WD40* transcription factor families, which together form the key MBW complex that promotes flower color formation (Kim et al., 2018; Jin et al., 2016). Hu et al. (2023) reported that the *MYB* transcription factor could bind to the promoter of *ANS* and increase its expression in *S. baicalensis*. Qi et al. (2020) demonstrated that *bHLH1* of *Paeonia suffruticosa* could transcriptionally activate the expression of *DFR* and *ANS* via direct binding to their promoters. *WD40* is primarily involved in the formation of the MBW complex and plays a regulatory role in anthocyanin biosynthesis (Gu et al., 2019). In addition to the MBW complex, transcription factors, such as *WRKY* and *NAC*, also play important roles in the regulation of anthocyanin biosynthesis (Verweij et al., 2016; Zhou et al., 2015). In this study, we retrieved 307 transcription factors, from RNA-seq data, including *OvMYB*, *OvbHLH*, *OvWD40*, *OvWRKY*, and *OvNAC*, which might be related to the synthesis of cyanidin. Compared with those in OvW, 70 transcription factors (28 *OvMYB*, 13 *OvbHLH*, 5 *OvWD40*, 14 *OvWRKY*, and 10 *OvNAC*) were differentially expressed in OvP. The expression of two transcription factors, *OvMYB* (OV09G037310) and *OvbHLH* (OV02G027910), significantly differed among the three groups of flowers and increased with increasing flower color, suggesting that these transcription factors may be the main regulatory factors associated with anthocyanin biosynthesis (**Supplemntary Table S3**). Interestingly, both these transcription factors were positively correlated with structural genes in the anthocyanidin biosynthetic pathway of *O. violaceus*, such as *OvANS*, *OvPAL*, *OvCHS*, *OvDFR*, and *OvUFGT*, according to Spearman’s correlation analysis (**Supplementary Figure S1**). Furthermore, the target genes of the transcription factors were predicted using FIMO software, which revealed that *OvMYB* might act on the *OvANS*, *OvPAL*, *OvDFR*, *OvF3’5’H*, and *OvF3’H* genes; *OvbHLH* might act on *OvDFR*, *OvUFGT*, *OvF3’H*, and *OvCHS*; and that *OvWD40* might act on *OvDFR*, *OvF3’H*, *OvCHS*, *OvPAL*, and *OvANS*.

**Figure 4:**
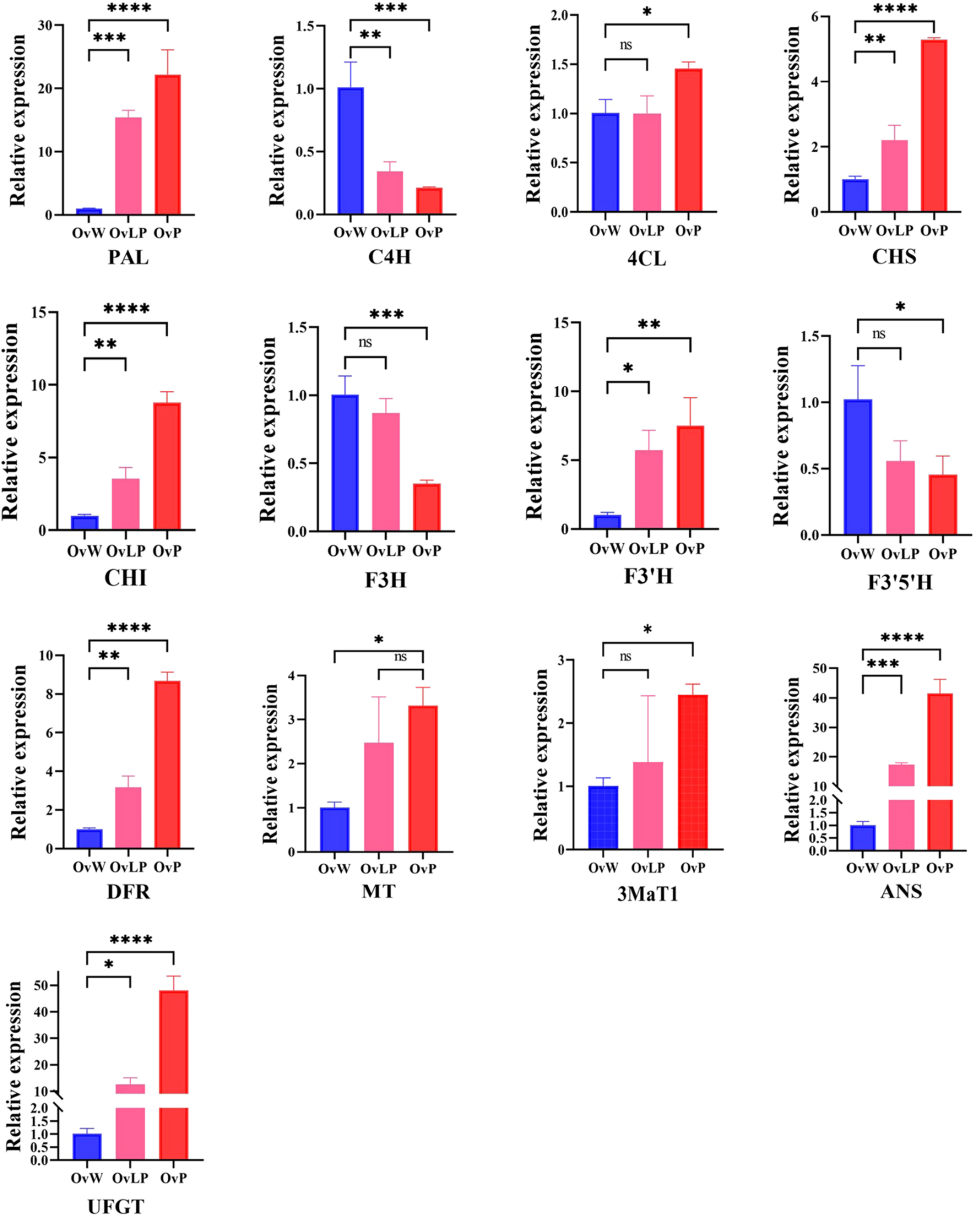
qPCR validation of key genes in the anthocyanin synthesis pathway of *O. violaceus*. The asterisk (*) for P < 0.05, (**) for P < 0.01, (***) for P < 0.001, and (****) for P < 0.0001. ns, no significant correlation.

To verify the reliability of the transcriptome information, we performed quantitative real-time PCR (qRT-PCR) to determine the expression of 13 DEGs involved in the synthetic pathway of anthocyanins. The results revealed that the expression of nine genes (*OvPAL*, *OvCHS*, *OvCHI*, *OvF3’H*, *OvDFR*, *Ov3MaT1*, *OvMT*, *OvUFGT*, and *OvANS*) increased as the flower color increased, whereas the expression of three genes (*OvC4H*, *OvF3H*, and *OvF3’5’H*) tended to decrease (**Figure 4**). The expression levels of OvUFGT, OvANS, and OvPAL were significantly higher in OvP than in OvW. Moreover, the expression of *Ov4CL* did not correlate with flower color. Overall, the qRT-PCR results were consistent with those of the transcriptome analysis, with the exception of the *OvC4H* gene, and further confirmed the correlation between the upregulated genes and flower color.

### 3.4 Comprehensive analysis of the transcriptome and metabolomics

**Figure 5:**
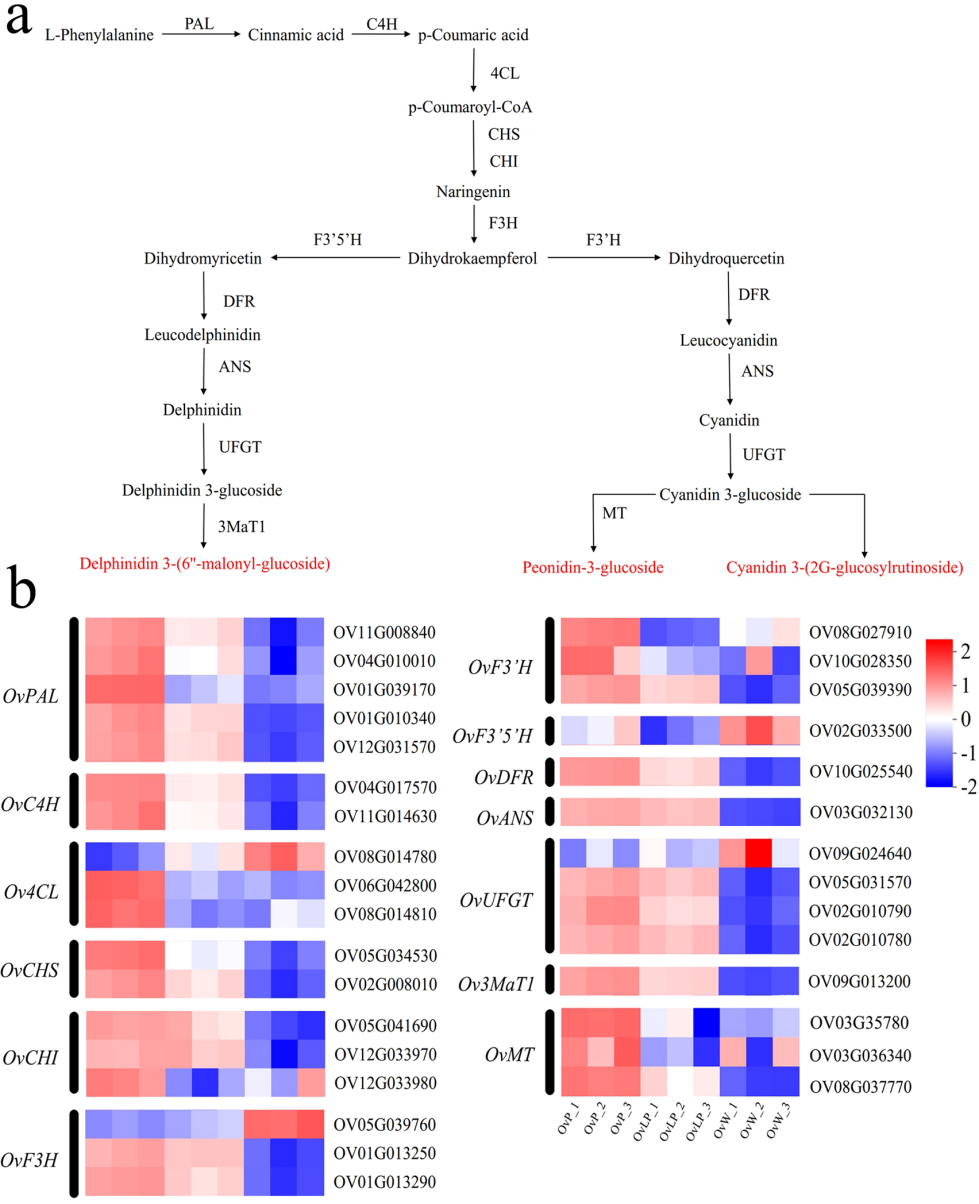
Studies on genes related to the anthocyanin synthesis pathway of *O. violaceus*. **(A)** Pathway diagram of anthocyanin synthesis in *O. violaceus*. *PAL*, phenylalanine ammonia lyase; *C4H*, cinnamate 4-hydroxylase; *4CL*, 4-coumarate–CoA ligase; *CHS*, chalcone synthase; *CHI*, chalcone isomerase; *F3H*, flavanone 3-hydroxylase; *DFR*, dihydroflavonol 4-reductase; *F3’H*, flavonoid 3’ hydroxylase; *F3’5’H*, flavonoid 3’,5’-hydroxylase; *ANS*, anthocyanidin synthase; *UFGT*, anthocyanidin 3-O-glucosyltransferase; *3MaT1*, anthocyanin 3-O-glucoside-6”-O-malonyltransferase; *MT*, methyltransferase. **(B)** Heatmap of differentially expressed genes associated with anthocyanin synthesis in *O. violaceus*.

Based on the metabolomic and transcription data, all structural genes involved in the anthocyanin biosynthetic pathway in *O. violaceus* were mapped (**Figure 5A**). As shown in **Figure 5B**, 32 prominent EDGs related to the anthocyanin biosynthetic pathway were selected for comparison between the three flower groups. The results of the analysis revealed that the expression of most DEGs increased with the purple color of the flowers. In particular, *OvANS*, which coverts colorless proanthocyanidins to colored anthocyanidins, was significantly upregulated in OvP and OvLP (**Figure 5B**). Moreover, *OvUFGT* was also highly expressed in OvP and OvLP, and could catalyze the glycosylation of anthocyanidins to form anthocyanins. The high expression of both key genes resulted in high anthocyanin production, which was consistent with the metabolomic data. Therefore, the metabolomic data revealed that three anthocyanins, peonidin-3-glucoside, delphinidin 3-(6”-malonyl-glucoside), and cyanidin 3-(2G-glucosylrutinoside), rather than the intermediates of the biosynthetic pathway, accumulated in OvP and OvLP (**Figure 5A**). Both metabolomic and transcriptomic data suggest that anthocyanin production affects the color of *O. violaceus* flowers. Finally, for a better understanding, we established a simple model of *O. violaceus* flower color change based on phenotypic, transcriptomic, and metabolomic data of the materials in this study (**Supplementary Figure S3**).

## 4 Conclusions

In this study, we investigated the molecular mechanisms of the three different flower colors of *O. violaceus* via a combination of metabolomics and transcriptomics. Metabolomic analysis revealed that the contents of three anthocyanins, peonidin-3-glucoside, delphinidin 3-(6”-malonyl-glucoside), and cyanidin 3-(2G-glucosylrutinoside), differed among the three flower types, and peonidin-3-glucoside and delphinidin 3-(6”-malonyl-glucoside) were positively correlated with color. Eighty-nine DEGs related to flavonoid biosynthesis were identified among the three distinct groups of flower samples via transcriptome analysis. Among these genes, the expression of *OvANS*, which is responsible for the biosynthesis of anthocyanins, significantly differed among the three flower types, which was further supported by qRT-PCR analysis. The results of the metabolomics and transcription analyses were consistent with the high expression of the three anthocyanins, suggesting that they were the dominant factors for the different flower colors of *O. violaceus*. Our study provides valuable information for investigating the genes and metabolism of the anthocyanin synthesis pathway in *O. violaceus*. Identification of the key genes responsible for the biosynthesis of these three anthocyanins lays the foundation for breeding *O. violaceus* with cauliflower color.

## Data Availability

The datasets presented in this study can be found in online repositories. The raw reads were submitted to NCBI SRA (Sequence Read Archive, http://www.ncbi.nlm.nih.gov/sra/ (accessed on 17 July 2024)) under the accession number PRJNA1136702.

## Supplementary material

Supplementary Figure 1 Correlation analysis of transcription factor expression. The red nodes represent transcription factors, the blue nodes represent structural genes, the red line represents a positive correlation, the blue line represents a negative correlation, the solid line represents the correlation between transcription factors and structural genes, and the dashed line represents the correlation between structural genes.

Supplementary Figure 2 Phylogenetic tree analysis of the *ANS* of *O. violaceus*.

Supplementary Table 1 qPCR primer sequence.

Supplementary Table 2 The transcription factors associated with the anthocyanin synthesis pathway of *O. violaceus* were significantly different between the OvP and OvW groups (VIP > 1 and *P* < 0.05).
