# Supplementary material for: Integrated transcriptomic and metabolomic analyses reveal the molecular mechanism of flower color differentiation in Orychophragmus violaceus
Source: Front Plant Sci. 2025 Feb 14;16:1509120. doi: 10.3389/fpls.2025.1509120 (PMC11868260; doi:10.3389/fpls.2025.1509120)
Supplement: Supplementary Figure 1 — Correlation analysis of transcription factor expression. The red nodes represent transcription factors, the blue nodes represent structural genes, the red line represents a positive correlation, the blue line represents a negative correlation, the solid line represents the correlation between transcription factors and structural genes, and the dashed line represents the correlation between structural genes. [file DataSheet1.zip › Supporting document (Revision)/Supplementary_Figure S1.docx]

Supplementary Material


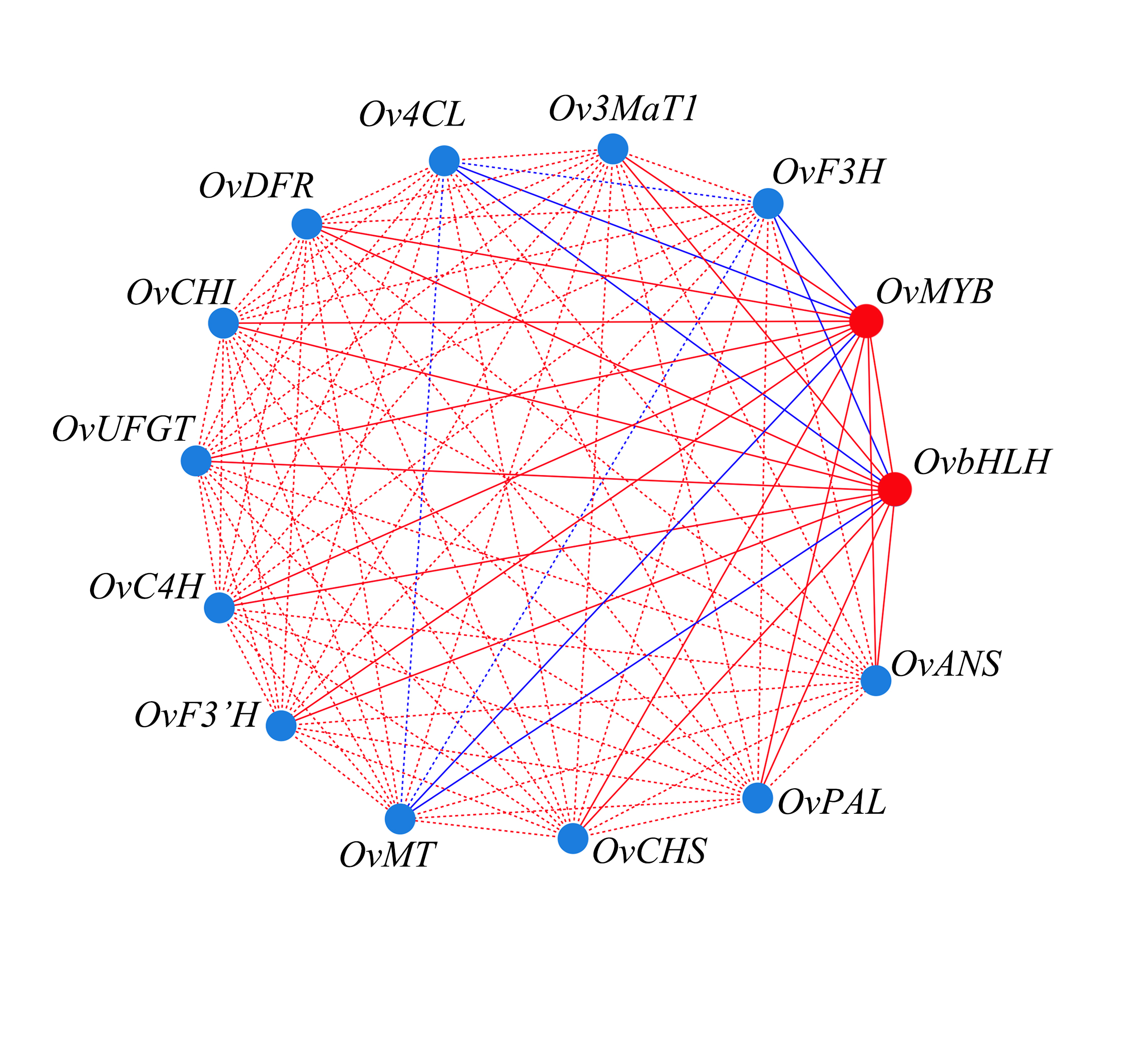


**Supplementary Figure S1.** Correlation analysis of transcription factor expression. The red nodes are transcription factors, the blue nodes are structural genes, the red line represents the positive correlation, the blue line represents the negative correlation, the solid line represents the correlation between transcription factors and structural genes, and the dashed line represents the correlation between structural genes.
