# Supplementary material for: Integrated transcriptomic and metabolomic analyses reveal the molecular mechanism of flower color differentiation in Orychophragmus violaceus
Source: Front Plant Sci. 2025 Feb 14;16:1509120. doi: 10.3389/fpls.2025.1509120 (PMC11868260; doi:10.3389/fpls.2025.1509120)
Supplement: Supplementary Figure 1 — Correlation analysis of transcription factor expression. The red nodes represent transcription factors, the blue nodes represent structural genes, the red line represents a positive correlation, the blue line represents a negative correlation, the solid line represents the correlation between transcription factors and structural genes, and the dashed line represents the correlation between structural genes. [file DataSheet1.zip › Supporting document (Revision)/Supplementary_Figure S2.docx]

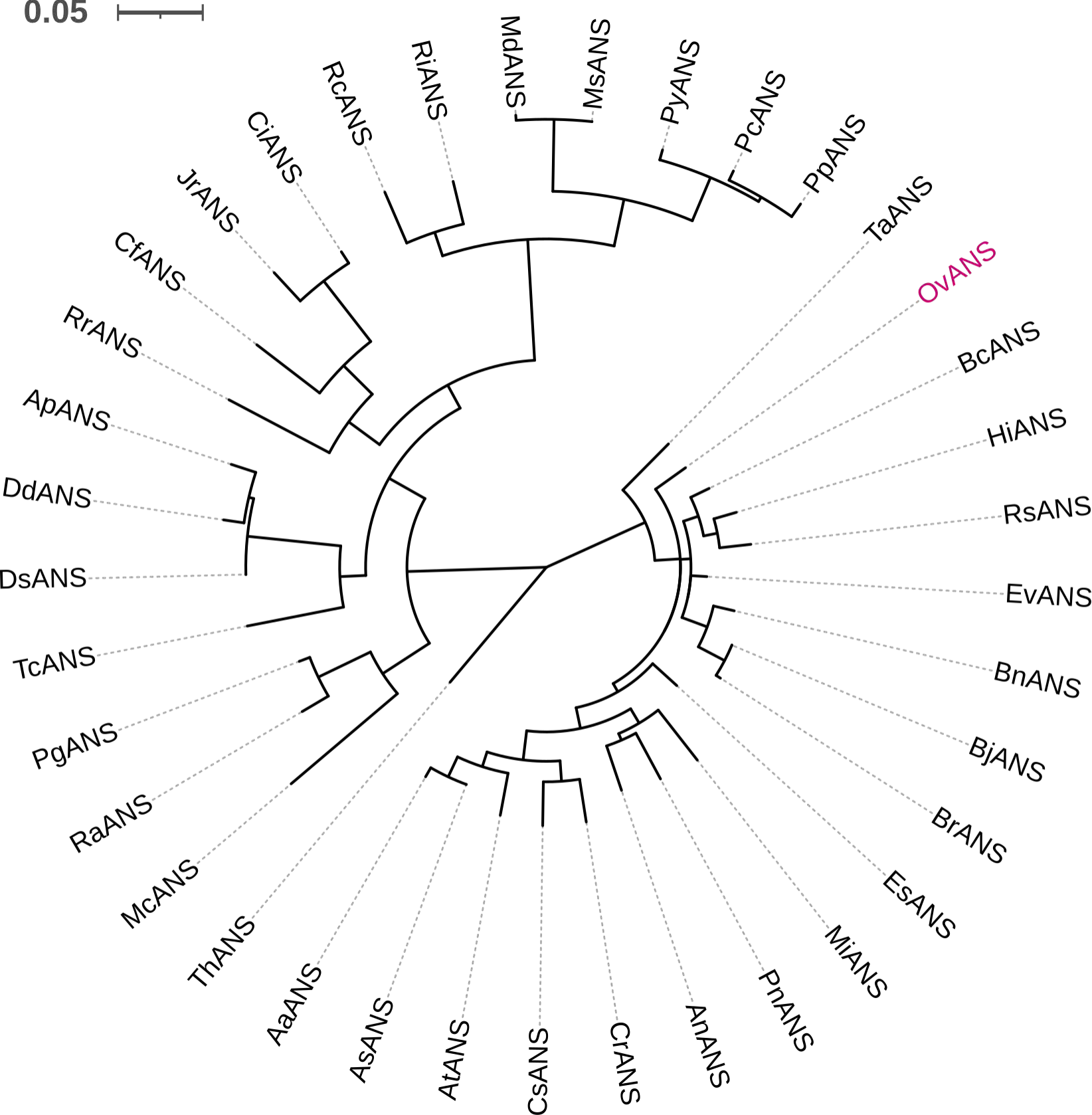
Supplementary Material

**Supplementary Figure S2.** Phylogenetic tree analysis of the *ANS* of *O. violaceus*. **EvANS:** *Eruca vesicaria subsp. Sativa***; EsANS:** *Eutrema salsugineum***; BrANS:** *Brassica rapa***; BcANS:** *Brassica carinata***; BjANS:** *Brassica juncea***; BnANS:** *Brassica napus***; RsANS:** *Raphanus sativus***; HiANS:** *Hirschfeldia incana***; TaANS:** *Thlaspi arvense***; AsANS:** *Arabidopsis suecica***; MiANS:** *Matthiola incana***; AaANS:** *Arabidopsis arenosa***; AnANS:** *Arabis nemorensis***; CsANS:** *Camelina sativa***; CrANS:** *Capsella rubella***; AtANS:** *Arabidopsis thaliana***; PnANS:** *Parrya nudicaulis***; ThANS:** *Tarenaya hassleriana***; PgANS:** *Psidium guajava***; RrANS:** *Rhamnella rubrinervis***; DsANS:** *Dipteronia sinensis***; RiANS:** *Rubus idaeus***; ApANS:** *Acer palmatum***; PpANS:** *Prunus persica***; McANS:** *Melastoma candidum***; RaANS:** *Rhodamnia argentea***; RcANS:** *Rosa chinensis***; CfANS:** *Carpinus fangiana***; PcANS:** *Prunus cerasifera***; CiANS:** *Carya illinoinensis***; MsANS:** *Malus sylvestris***; JrANS:** *Juglans regia***; TcANS:** *Theobroma cacao***; PyANS:** *Prunus yedoensis var. nudiflora***; DdANS:** *Dipteronia dyeriana***; MdANS:** *Malus domestica*.
