# Supplementary material for: Integrated transcriptomic and metabolomic analyses reveal the molecular mechanism of flower color differentiation in Orychophragmus violaceus
Source: Front Plant Sci. 2025 Feb 14;16:1509120. doi: 10.3389/fpls.2025.1509120 (PMC11868260; doi:10.3389/fpls.2025.1509120)
Supplement: Supplementary Figure 1 — Correlation analysis of transcription factor expression. The red nodes represent transcription factors, the blue nodes represent structural genes, the red line represents a positive correlation, the blue line represents a negative correlation, the solid line represents the correlation between transcription factors and structural genes, and the dashed line represents the correlation between structural genes. [file DataSheet1.zip › Supporting document (Revision)/Supplementary_Figure S3.docx]

Supplementary Material

**
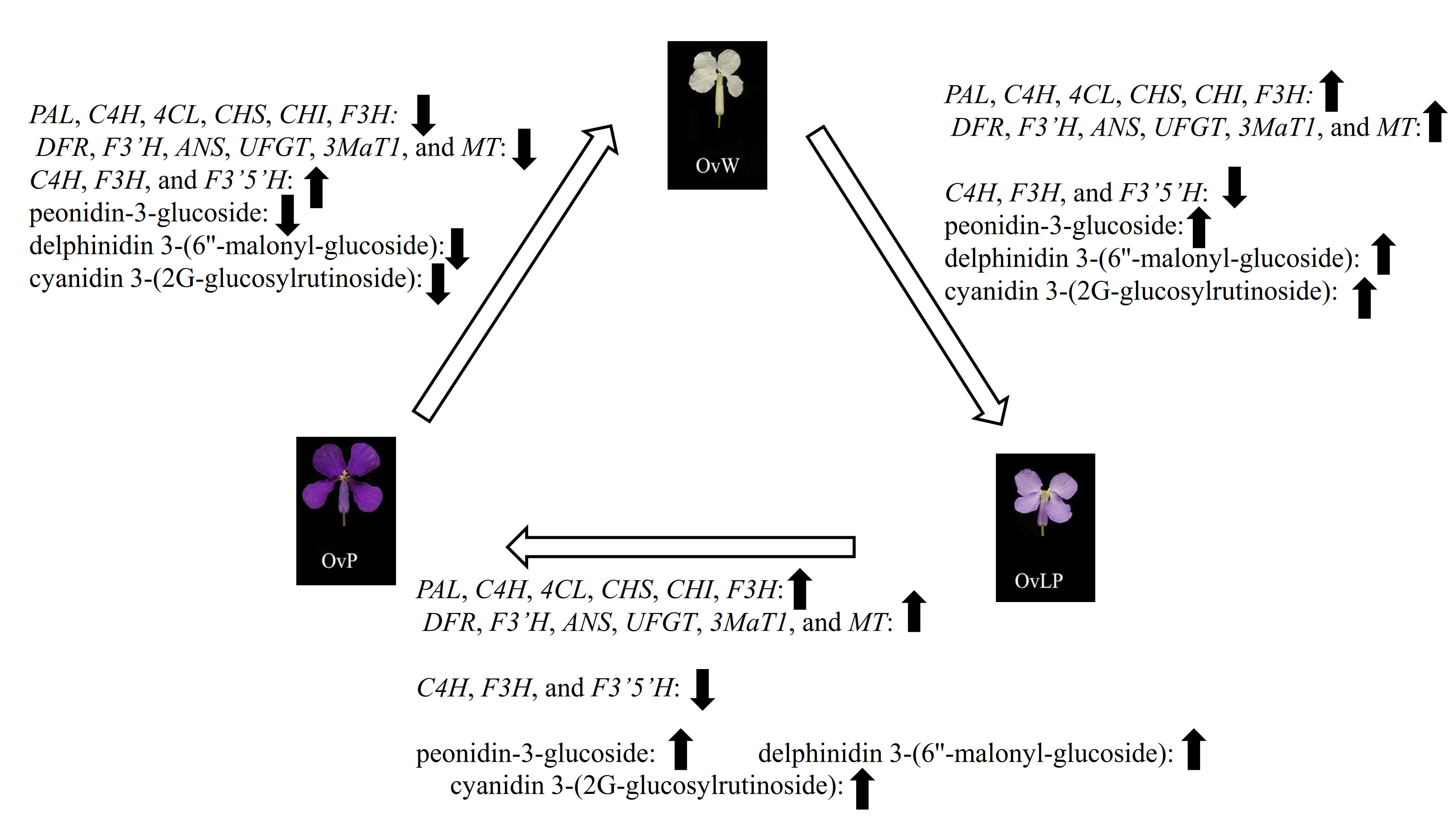
**

**Supplementary Figure S3.** Molecular mechanism model of *O. violaceus* flower color differentiation.
